# Correction: Potassium 2-methoxy-4-vinylphenolate: a novel hit exhibiting quorum-sensing inhibition in *Pseudomonas aeruginosa via* LasIR/RhlIR circuitry

**DOI:** 10.1039/c9ra90095k

**Published:** 2019-12-18

**Authors:** Mayank D. Shah, Prashant S. Kharkar, Niteshkumar U. Sahu, Zoya Peerzada, Krutika B. Desai

**Affiliations:** Sunandan Divatia School of Science, SVKM’s NMIMS (Deemed to be University) Mumbai 400056 India; Shobhaben Pratapbhai Patel School of Pharmacy and Technology Management, SVKM’s NMIMS (Deemed to be University) Mumbai India; Mithibai College of Arts & Science & Amrutben Jivanlal College of Commerce & Economics Mumbai 400056 India krutika.desai@mithibai.ac.in; Department of Pharmaceutical Sciences and Technology, Institute of Chemical Technology Matunga Mumbai 400 019 India

## Abstract

Correction for ‘Potassium 2-methoxy-4-vinylphenolate: a novel hit exhibiting quorum-sensing inhibition in *Pseudomonas aeruginosa via* LasIR/RhlIR circuitry’ by Mayank D. Shah *et al.*, *RSC Adv.*, 2019, **9**, 40228–40239.

The authors regret that one of the affiliations (affiliation *b*) was incorrectly shown in the original manuscript. The authors would like to add affiliation *d* as the current affiliation for Prashant S. Kharkar. The corrected list of affiliations is as shown above.

The Royal Society of Chemistry apologises for these errors and any consequent inconvenience to authors and readers.

## Supplementary Material

